# Small Molecule Library Synthesis Using Segmented Flow

**DOI:** 10.3390/molecules16119161

**Published:** 2011-11-02

**Authors:** Christina M. Thompson, Jennifer L. Poole, Jeffrey L. Cross, Irini Akritopoulou-Zanze, Stevan W. Djuric

**Affiliations:** 1 Medinical Chemistry Technologies, Abbott Laboratories, Global Pharmaceutical Research and Development, 100 Abbott Park Road, Abbott Park, IL 60064, USA; 2 Department of Chemistry at KU, 1251 Wescoe hall Drive, 2010 Marlott Hall, Lawrence, KA 66045, USA

**Keywords:** segmented flow, library synthesis

## Abstract

Flow chemistry has gained considerable recognition as a simple, efficient, and safe technology for the synthesis of many types of organic and inorganic molecules ranging in scope from large complex natural products to silicon nanoparticles. In this paper we describe a method that adapts flow chemistry to the synthesis of libraries of compounds using a fluorous immiscible solvent as a spacer between reactions. The methodology was validated in the synthesis of two small heterocycle containing libraries. The reactions were performed on a 0.2 mmol scale, enabling tens of milligrams of material to be generated in a single 200 μL reaction plug. The methodology allowed library synthesis in half the time of conventional microwave synthesis while maintaining similar yields. The ability to perform multiple, potentially unrelated reactions in a single run is ideal for making small quantities of many different compounds quickly and efficiently.

## 1. Introduction

Flow chemistry reactors have received increased attention over the last decade as flexible platforms for organic synthesis. Their modular and simple designs have been adapted to a wide range of applications, from the synthesis of small molecules [1,2] and complex natural products [3,4,5,6], to nanoparticle synthesis [7,8]. Among their many benefits are excellent heat and mass transfer capabilities, allowing for concise temperature control at very high temperatures for increasing reaction rates, and also at low temperatures for increased control when performing exothermic reactions [9,10,11]. The inherent closed nature of flow systems enables very high pressures under superheating conditions and containment of noxious or toxic reagents. The small reaction volume has enabled reactions that were once avoided, as only a small amount of reactive intermediates is present at a given time [12]. The added ability to change temperature, residence time, and relative stoichiometry quickly and easily during a reaction through a computer control makes flow reactor systems excellent for reaction development and library preparation.

Plug, or segmented flow, which uses either a gaseous bubble or an immiscible solvent spacer to separate “plugs” of desired reactions within a continuous flow machine, have also found a wide range of applications. For example, Wheeler *et al*. reported a plug flow approach for reaction optimization using a range of organic solvents separated by fluorinated spacers [13,14]. Plug flow has also been successfully applied to protein crystallization [15], measurement of reaction kinetics [16], and high-throughput screening [17,18,19].

Our goal was to adapt the modular and high-throughput nature of flow synthesis technology for use in the generation of small molecule libraries using a single generic instrument set up. We felt that this method should be equal to or more efficient than traditional solution phase chemistry. In this context we describe the synthesis of two different heterocyclic libraries in yields comparable to traditional techniques. The reactions were performed on a 0.2 mmol scale and were completed in half the time of traditional serial microwave synthesis using a continuous process. By encapsulating each individual library member inside a single organic droplet, and separating those with a fully immiscible fluorinated solvent, we gained the ability to spatially separate library members along the length of a reactor tube. We have utilized the synthesis of thiazoles and pyrazoles to develop this methodology.

## 2. Results and Discussion

**Figure 1:**
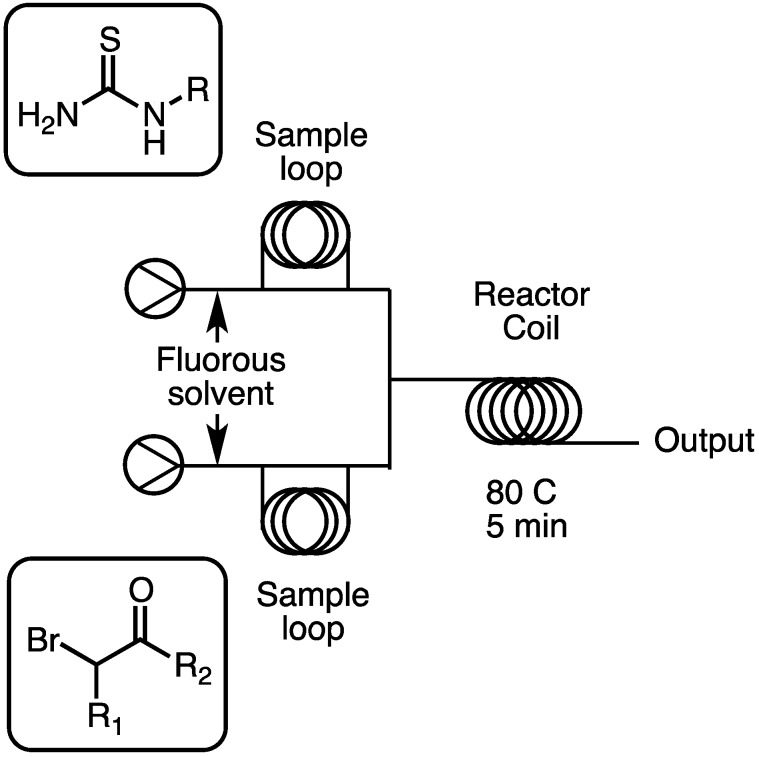
Diagram of the flow reactor set up for library synthesis.

The commercially available Uniqsis mesoscale flow machine [18] was employed for the development of this methodology. The flow reactor was composed of two HPLC pumps that were each responsible for a solvent and a reagent stream, and a variable temperature flow coil. The two required starting materials for a given library member were loaded into 100 μL PTFE injection loops, one per pump (Figure 1). These injection loops served a dual purpose of metering the correct amount of reagent for the reaction, and also as a temporary holding port. The carrier liquid for both pumps was a commercially available fully fluorinated solvent, F-40. When switched to inject, fluorinated solvent drove the liquid out of both reagent loops simultaneously, where they meet at a T-joint to form a single cohesive 200 μL reaction droplet.

It was found that solvents with higher surface tension formed more cohesive droplets than did solvents of lower surface tension. Solvents such as MeOH, DMF, and water formed the most stable droplets, while solvents such as acetonitrile, EtOAc, and CH_2_Cl_2_ tended to form chains of small droplets. Droplet stability was also influenced by the flow rate, with 1.3 mL/min being the minimal rate required for reliable droplet formation. The total volume of the droplets was controlled by the size of the injector loops and could be varied for specific applications.

The study was conducted on a 0.2 mmol scale, with 0.2 mmol of each reagent being dissolved in 0.1 mL of DMF. These were then serially loaded into the injection loops and injected in a standard 4 × 5 matrix to yield the desired 20 member libraries shown in Table 1 and Table 2. Initially, a small amount of contamination from one reaction to the next was observed in the LCMS trace of the crude libraries. To eliminate this problem, each injection of a library compound was followed by an injection of pure DMF.

**Table 1:** Twenty member thiazole library synthesized in droplets using a continuous flow syntheisizer. 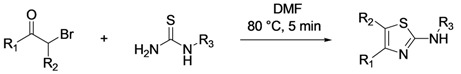

Compound	R_1_	R_2_	R_3_	Yield (%) ^a^
1-1	*p*-CNPh	H	allyl	42
1-2	*p*-CNPh	H	Ph	48
1-3	*p*-CNPh	H	*m*-FPh	49
1-4	*p*-CNPh	H	CH_3_	48
1-5	R_1_-C_6_H_4_-2-CH_2_-R_2_		allyl	12
1-6	R_1_-C_6_H_4_-2-CH_2_-R_2_		Ph	34
1-7	R_1_-C_6_H_4_-2-CH_2_-R_2_		*m*-FPh	24
1-8	R_1_-C_6_H_4_-2-CH_2_-R_2_		CH_3_	53
1-9	*t*Bu	H	allyl	50
1-10	*t*Bu	H	Ph	*40*
1-11	*t*Bu	H	*m*-FPh	49
1-12	*t*Bu	H	CH_3_	36
1-13	*p*-FPh	H	allyl	63
1-14	*p*-FPh	H	Ph	31
1-15	*p*-FPh	H	*m*-FPh	46
1-16	*p*-FPh	H	CH_3_	55
1-17	Ph	CH_3_	allyl	41
1-18	Ph	CH_3_	Ph	40
1-19	Ph	CH_3_	*m*-FPh	31
1-20	Ph	CH_3_	CH_3_	61

^a^ Combined chemical yield after purification.

**Table 2:** Twenty member pyrazole library synthesized in droplets using a continuous flow syntheisizer. 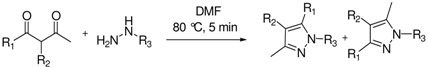

Compound	R_1_	R_2_	R_3_	Yield ^a^	Regioisomers ^b^
2(a+b)-1	R_1_-(CH_2_)_3_-R_2_		*m,m*-ClPh	9	89:11
2(a+b)-2	CF_3_	H	*m,m*-ClPh	15	100
2(a+b)-3	CH_3_	CH_3_	*m,m*-ClPh	26	-
2(a+b)-4	Ph	H	*m,m*-ClPh	23	60:40
2(a+b)-5	R_1_-(CH_2_)_3_-R_2_		(CH_2_)_2_OH	50	93:7
2(a+b)-6	CF_3_	H	(CH_2_)_2_OH	17	100
2(a+b)-7	CH_3_	CH_3_	(CH_2_)_2_OH	37	-
2(a+b)-8	Ph	H	(CH_2_)_2_OH	79	72:28
2(a+b)-9	R_1_-(CH_2_)_3_-R_2_		Ph	32	100
2(a+b)-10	CF_3_	H	Ph	38	100
2(a+b)-11	CH_3_	CH_3_	Ph	33	-
2(a+b)-12	Ph	H	Ph	29	100
2(a+b)-13	R_1_-(CH_2_)_3_-R_2_		Me	43	100
2(a+b)-14	CF_3_	H	Me	0	-
2(a+b)-15	CH_3_	CH_3_	Me	44	-
2(a+b)-16	Ph	H	Me	21	74:26
2(a+b)-17	R_1_-(CH_2_)_3_-R_2_		Cy	40	100
2(a+b)-18	CF_3_	H	Cy	23	100
2(a+b)-19	CH_3_	CH_3_	Cy	34	-
2(a+b)-20	Ph	H	Cy	26	100

^a^ Combined chemical yield after purification; ^b^ Ratio of regioisomers.

The first library synthesized was a thiazole library. The reactions were performed at 80 °C and had a residency time of 5 min. For this library, the average purified yield was 53%, with a range from 12%–63% (Table 1).

This method was then applied to a twenty member pyrazole library. The yields for this library ranged from 9%–79%, with one library member failing to produce any of the desired product. Mixtures of regioisomers were obtained in cases where unsymmetric diketones were employed. The major regioisomer observed was typically the one obtained from the reaction of hydrazines with the less hindered carbonyl group of the diketone. When R_1_ is CF_3_, a single regioisomer is obtained. Despite the lower yields, the library produced an average of 12 milligrams of purified product per compound, more than sufficient for primary bioassays.

To establish if the flow method provided similar chemical yields to that of established microwave methods, several examples from both libraries representing a range of chemical yields were then resynthesized in a microwave on the same scale and under the same temperature and time conditions as the flow reactions. Good correlation between the two methodologies was observed (Table 3).

**Table 3:** Comparison between microwave and flow yields. Reactions were performed on 0.2 mmol scale. All yields are purified yields.

Compound	Microwave Yield	Flow Yield	Batch Yield
1-7	34%	24%	28%
1-13	54%	63%	59%
1-17	51%	41%	46%
2-5	54%	50%	48%
2-10	32%	38%	33%
2-14	0%	0%	0%

## 3. Experimental

### 3.1. General

All reagents and solvents were supplied from Aldrich and were used without further purification. ^1^H-NMR spectra were recorded at ambient temperatures at either 400 MHz or 500 MHz on Varian 400-MR, Inova or VNMRS spectrometers. Deuterated solvents were used as received from Sigma-Aldrich and data is reported as follows; chemical shift in ppm from tetramethylsilane as an internal standard, multiplicity (s = singlet, d = doublet, dd = doublet of doublets, ddd = doublet of doublet of doublets, bd = broad doublet, t = triplet, dt = doublet of triplets, td = triplet of doublets, and m = multiplet), integration value. ^13^C-NMR were recorded at ambient temperature at 100 MHz or 125 MHz on Varian 400-MR or Inova 500 spectrometers and data is reported as follows; chemical shift in ppm from tetramethylsilane as an internal standard. Regioisomers were assigned based correlations observed in 2-dimensional rotating frame Overhauser effect spectroscopy (ROESY) experiments. Microwave experiments were conducted in a Biotage Initiator microwave, a single-mode 400 W instrument that uses an infrared temperature sensor.

### 3.2. Standard Protocol for Library Synthesis

Stock solutions of each desired starting material were first created by adding 2 mmol of a given starting material to a 20 mL scintillation vial. To each of these vials was then added 2 mL of dry DMF, and the vials were then sonicated to form clear solutions. For each library member, 100 μL of stock solution from each of the required building blocks was loaded into a metering loop of known volume on a Uniqsis flow system. The machine then injected both metering loops to form a single cohesive droplet that was then flowed at 1.3 mL/min through an 80 °C heated coil, giving a final reaction time of 5 min. One minute after the library member was injected, a blank plug of 200 mL of DMF was added to the machine, and both the library droplet and the wash droplet were collected into tubes. The DMF layer was then removed, the DMF was evaporated under warm nitrogen, and the resulting residue was purified by preparative HPLC on a Waters Symmetry C8 column (25 mm × 100 mm, 7 μm particle size) using a gradient of 10% to 100% acetonitrile: 0.1% aqueous TFA over 8 min (10 min run time) at a flow rate of 40 mL/min to give the desired compound. The pyrrazole library was repeated in triplicate, and gave a 5.1% standard deviation in yields.


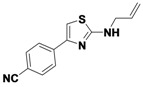

*4-(2-(Allylamino)thiazol-4-yl)benzonitrile* (**1-1**). ^1^H-NMR (500 MHz, DMSO) δ 8.00 (d, *J* = 8.4, 3H), 7.83 (d, *J* = 8.3, 2H), 7.41 (s, 1H), 5.94 (ddd, *J* = 22.5, 10.5, 5.4, 1H), 5.28 (d, *J* = 17.2, 1H), 5.14 (d, *J* = 10.2, 1H), 3.93 (d, *J* = 19.5, 2H). ^13^C-NMR (101 MHz, DMSO) δ 168.36, 147.98, 138.82, 134.76, 132.41, 126.17, 119.04, 115.97, 109.33, 105.09, 46.61.MS (DCI+) *m/z* 242.1 [M+H]^+^. 42% yield.


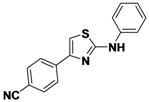

*4-(2-(Phenylamino)thiazol-4-yl)benzonitrile* (**1-2**). ^1^H-NMR (500 MHz, DMSO) δ 10.36 (s, 1H), 8.11 (d, *J* = 8.4, 2H), 7.90 (d, *J* = 8.4, 2H), 7.72 (d, *J* = 7.9, 2H), 7.66 (s, 1H), 7.36 (t, *J* = 7.9, 2H), 6.99 (t, *J* = 7.3, 1H). ^13^C-NMR (126 MHz, DMSO) δ 163.37, 148.33, 140.95, 138.54, 132.72, 129.03, 126.23, 121.43, 119.01, 116.91, 109.58, 106.87. MS (DCI+) *m/z* 278.3 [M+H]^+^. 48% yield.


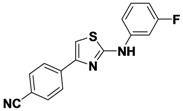

*4-(2-(3-Fluorophenylamino)thiazol-4-yl)benzonitrile* (**1-3**). ^1^H-NMR (500 MHz, DMSO) δ 10.61 (s, 1H), 8.10 (d, *J* = 8.3, 2H), 7.92 (d, *J* = 8.3, 2H), 7.78 (d, *J* = 11.8, 1H), 7.72 (s, 1H), 7.44–7.27 (m, 3H), 6.80 (m, 1H). ^13^C-NMR (126 MHz, DMSO) δ 162.96, 162.54 (d, *J* = 241), 148.36, 142.55 (d, *J* = 11), 138.39, 132.78, 130.58 (d, *J* = 10), 126.20, 118.98, 112.83 (d, *J* = 2), 109.71, 107.65 (d, *J* = 21), 107.51, 103.58 (d, *J* = 27). MS (DCI+) *m/z* 296.3 [M+H]^+^. 49% yield.


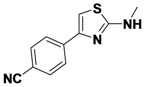

*4-(2-(Methylamino)thiazol-4-yl)benzonitrile* (**1-4**). ^1^H-NMR (500 MHz, DMSO) δ 8.01 (d, *J* = 8.2, 2H), 7.90 (d, *J* = 8.2, 2H), 7.39 (s, 1H), 3.15 (s, 3H). ^13^C-NMR (101 MHz, DMSO) δ 169.48, 147.79, 138.66, 132.53, 126.22, 119.02, 109.36, 104.88, 31.01. MS (DCI+) *m/z* 216.3 [M+H]^+^. 48% yield.


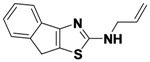

*N-Allyl-8H-indeno[1,2-d]thiazol-2-*amine (**1-5**). ^1^H-NMR (500 MHz, DMSO) δ 7.47 (dd, *J* = 13.3, 7.4, 2H), 7.31 (t, *J* = 7.5, 1H), 7.18 (t, *J* = 7.4, 1H), 5.95 (ddt, *J* = 17.0, 10.4, 5.3, 1H), 5.41–5.22 (m, 1H), 5.17 (dd, *J* = 10.2, 1.4, 1H), 4.02 (s, 1H). ^13^C-NMR (126 MHz, DMSO) δ 173.04, 145.42, 136.79, 134.33, 126.65, 124.71, 124.34, 122.51, 117.68, 116.17, 46.72, 32.48. MS (DCI+) *m/z* 229.1 [M+H]^+^. 12% yield.


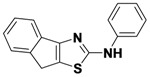

*N-Phenyl-8H-indeno[1,2-d]thiazol-2-amine* (**1-6**). ^1^H-NMR (500 MHz, DMSO) δ 10.33 (s, 1H), 7.73 (d, *J* = 7.9, 2H), 7.54 (dd, *J* = 17.7, 7.4, 2H), 7.37–7.31 (m, *J* = 7.5, 3.9, 3H), 7.21 (t, *J* = 9.5, 5.4, 1H), 6.97 (t, *J* = 7.3, 1H), 3.81 (s, 2H). ^13^C-NMR (126 MHz, DMSO) δ 167.47, 156.16, 145.40, 141.05, 137.37, 129.00, 126.71, 124.78, 124.40, 121.26, 117.76, 116.91, 32.29. MS (DCI+) *m/z* 265.1 [M+H]^+^. 34% yield.


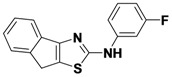

*N-(3-fluorophenyl)-8H-indeno[1,2-d]thiazol-2-amine* (**1-7**). ^1^H-NMR (500 MHz, DMSO) δ 7.81 (d, *J* = 7.3, 1H), 7.58 (d, *J* = 7.5, 1H), 7.53 (d, *J* = 7.4, 1H), 7.38–7.32 (m, 3H), 7.22 (t, *J* = 7.5, 1H), 6.81–6.75 (m, 1H), 3.83 (s, 2H). ^13^C-NMR (101 MHz, DMSO) δ 166.95, 162.60 (d, *J* = 241), 156.14, 145.38, 142.67 (d, *J* = 11), 137.21, 130.51 (d, *J* = 10), 126.76, 125.18, 124.83, 124.53, 117.84, 112.78 (d, *J* = 2), 107.43 (d, *J* = 21), 103.57 (d, *J* = 27), 32.32. MS (DCI+) *m/z* 283.3 [M+H]^+^. 24% yield.


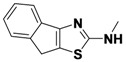

*N-methyl-8H-indeno[1,2-d]thiazol-2-amine* (**1-8**). ^1^H-NMR (500 MHz, DMSO) δ 7.51–7.44 (m, 2H), 7.32 (t, *J* = 7.5, 1H), 7.19 (t, *J* = 7.5, 1H), 3.72 (s, *J* = 18.8, 2H), 2.96 (s, *J* = 5.4, 3H). ^13^C-NMR (101 MHz, DMSO) δ 173.89, 145.39, 136.38, 126.67, 124.73, 124.47, 122.08, 117.72, 32.61, 31.19. MS (DCI+) *m/z* 203.3 [M+H]^+^. 53% yield.


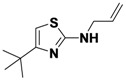

*N-Allyl-4-tert-butylthiazol-2-amine* (**1-9**). ^1^H-NMR (500 MHz, DMSO) δ 6.46 (s, 1H), 5.88 (m, 1H), 5.31 (d, *J* = 17.2, 1H), 5.23 (d, *J* = 10.3, 1H), 3.96 (s, *J* = 4.1, 2H), 1.24 (s, 9H). ^13^C-NMR (101 MHz, DMSO) δ 169.78, 149.34, 131.76, 117.94, 100.05, 48.11, 33.25, 28.39. MS (DCI+) *m/z* 197.1 [M+H]^+^. 50% yield.


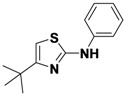

*4-tert-Butyl-N-phenylthiazol-2-amine* (**1-10**). ^1^H-NMR (500 MHz, DMSO) δ 10.08 (s, 1H), 7.68–7.54 (m, 2H), 7.30 (t, *J* = 7.7, 2H), 6.92 (t, *J* = 7.3, 1H), 6.41 (s, 1H), 1.31 (s, 9H). ^13^C-NMR (101 MHz, DMSO) δ 162.93, 160.86, 141.29, 128.96, 121.15, 116.87, 99.21, 34.31, 29.54. MS (DCI+) *m/z* 233.2 [M+H]^+^. 40% yield.


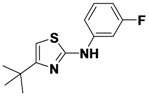

*4-tert-Butyl-N-(3-fluorophenyl)thiazol-2-amine* (**1-11**). ^1^H-NMR (500 MHz, DMSO) δ 10.33 (s, 1H), 7.82–7.65 (m, 1H), 7.41–7.16 (m, 2H), 6.71–6.65 (m, 1H), 6.57–6.38 (m, 1H), 1.27 (s, 9H). ^13^C-NMR (126 MHz, DMSO) δ 162.56 (d, *J* = 241), 162.04, 161.53, 143.12 (d, *J* = 12), 130.36 (d, *J* = 10), 112.44, 106.95 (d, *J* = 21), 103.24 (d, *J* = 27), 99.98, 34.39, 29.59. MS (DCI+) *m/z* 251.1 [M+H]^+^. 49% yield.


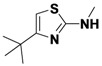

*4-tert-Butyl-N-methylthiazol-2-amine* (**1-12**). ^1^H-NMR (500 MHz, DMSO) δ 6.50 (s, 1H), 2.97 (s, 3H), 1.32 (s, 9H). ^13^C-NMR (101 MHz, DMSO) δ 170.27, 162.27, 148.86, 100.12, 33.19, 28.34. MS (DCI+) *m/z* 171.3 [M+H]^+^. 36% yield.


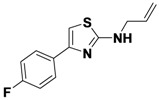

*N-Allyl-4-(4-fluorophenyl)thiazol-2-amine* (**1-13**). ^1^H-NMR (500 MHz, DMSO) δ 7.90–7.74 (m, 2H), 7.21 (t, *J* = 8.8, 2H), 7.06 (s, 1H), 5.93–5.85 (m, 1H), 5.28 (d, *J* = 8.8, 2H), 5.15 (d, *J* = 10.3, 2H), 3.95 (d, *J* = 5.2, 1H). ^13^C-NMR (101 MHz, DMSO) δ 168.42, 161.53 (d, *J* = 244), 147.87, 134.65, 130.91 (d, *J* = 3), 127.65 (d, *J* = 8), 116.05, 115.29 (d, *J* = 21), 100.96, 46.77. MS (DCI+) *m/z* 235.2 [M+H]^+^. 63% yield.


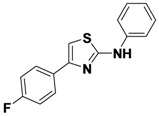

*4-(4-Fluorophenyl)-N-phenylthiazol-2-amine* (**1-14**). ^1^H-NMR (500 MHz, DMSO) δ 10.27 (s, 1H), 8.03–7.89 (m, 2H), 7.72 (d, *J* = 8.3, 2H), 7.42–7.30 (m, 2H), 7.24–7.3 (t, *J* = 7.25, 2H), 6.98 (t, *J* = 6.98, 1H). ^13^C-NMR (101 MHz, DMSO) δ 163.17, 161.60 (d, *J* = 244), 149.01, 141.14, 131.13 (d, *J* = 3), 128.99, 127.62 (d, *J* = 8), 121.22, 116.80, 115.46 (d, *J* = 21), 102.63. MS (DCI+) *m/z* 271.3 [M+H]^+^. 31% yield.


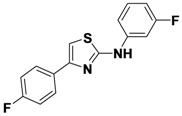

*N-(3-Fluorophenyl)-4-(4-fluorophenyl)thiazol-2-amine* (**1-15**). ^1^H-NMR (500 MHz, DMSO) δ 10.52 (s, 1H), 7.95 (dd, *J* = 8.4, 5.7, 2H), 7.79 (d, *J* = 11.0, 1H), 7.42–7.32 (m, 3H), 7.29 (t, *J* = 8.8, 2H), 6.78 (m, 1H). ^13^C-NMR (126 MHz,z DMSO) δ 162.73, 162.55 (d, *J* = 241), 161.65 (d, *J* = 244), 149.04, 142.73 (d, *J* = 11), 130.98 (d, *J* = 3), 130.52 (d, *J* = 10), 127.59 (d, *J* = 8), 115.55 (d, *J* = 21), 112.70 (d, *J* = 2), 107.41 (d, *J* = 21), 103.45 (d, *J* = 27), 103.34. MS (DCI+) *m/z* 289.4 [M+H]^+^. 46% yield.


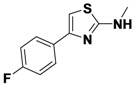

*4-(4-Fluorophenyl)-N-methylthiazol-2-amine* (**1-16**) ^1^H-NMR (500 MHz, DMSO) δ 7.90–7.80 (m, 2H), 7.28–7.18 (m, 2H), 7.23 (s, 1H), 2.91 (s, 3H). ^13^C-NMR (126 MHz, DMSO) δ 169.45, 161.52 (d, *J* = 244), 148.07, 130.92, 127.65 (d, *J* = 8), 115.28 (d, *J* = 22), 100.72. MS (DCI+) *m/z* 209.2 [M+H]^+^. 55% yield.


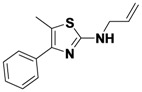

*N-Allyl-5-methyl-4-phenylthiazol-2-amine* (**1-17**). ^1^H-NMR (500 MHz, DMSO) δ 7.55 (d, *J* = 8.1, 2H), 7.46 (t, *J* = 7.6, 2H), 7.38 (t, *J* = 7.3, 1H), 5.91 (dtd, *J* = 15.8, 10.5, 5.3, 1H), 5.30 (d, *J* = 10.2, 1H), 5.18 (d, *J* = 10.3, 1H), 3.95 (s, 2H), 2.31 (s, 3H). ^13^C-NMR (101 MHz, DMSO) δ 166.55, 136.86, 132.60, 129.95, 128.89, 128.66, 117.39, 114.58, 114.49, 47.48, 11.66. MS (DCI+) *m/z* 231.2 [M+H]^+^. 41% yield.


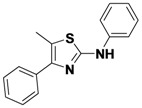

*5-Methyl-N,4-diphenylthiazol-2-amine* (**1-18**). ^1^H-NMR (500 MHz, DMSO) δ 10.05 (s, 1H), 7.66 (dd, *J* = 12.6, 8.3, 4H), 7.46 (t, *J* = 7.6, 2H), 7.37–7.26 (m, 3H), 6.92 (td, *J* = 7.4, 0.9, 1H), 2.43 (s, 3H). ^13^C-NMR (101 MHz, DMSO) δ 159.59, 144.82, 141.21, 134.86, 129.00, 128.36, 128.05, 127.21, 121.21, 116.96, 116.30, 11.93. MS (DCI+) *m/z* 267.2 [M+H]^+^. 40% yield.


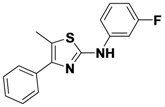

*N-(3-Fluorophenyl)-5-methyl-4-phenylthiazol-2-amine* (**1-19**). ^1^H-NMR (500 MHz, DMSO) δ 10.30 (s, 1H), 7.74 (d, *J* = 12.2, 1H), 7.67 (d, *J* = 8.0, 2H), 7.47 (t, *J* = 7.6, 2H), 7.40–7.24 (m, 3H), 6.82–6.65 (m, 1H), 2.44 (s, 3H). ^13^C-NMR (126 MHz, DMSO) δ 162.54 (d, *J* = 241), 158.71, 145.30, 142.90 (d, *J* = 12 Hz), 134.98, 130.40 (d, *J* = 10), 128.36, 127.92, 127.17, 117.04, 112.51 (d, *J* = 2), 107.02 (d, *J* = 21), 103.25 (d, *J* = 27), 11.91.MS (DCI+) *m/z* 285.4 [M+H]^+^. 31% yield.


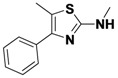

*N,5-Dimethyl-4-phenylthiazol-2-amine* (**1-20**). ^1^H-NMR (500 MHz, DMSO) δ 7.55 (d, *J* = 7.5, 2H), 7.49 (t, *J* = 7.6, 2H), 7.43 (t, *J* = 7.2, 1H), 2.96 (s, 3H), 2.27 (s, 3H). ^13^C-NMR (101 MHz, DMSO) δ 166.86, 134.06, 129.42, 128.98, 128.72, 128.28, 114.50, 31.96, 11.61. MS (DCI+) *m/z* 205.2 [M+H]^+^. 61% yield.


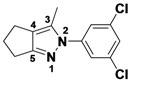

*2-(3,5-Dichlorophenyl)-3-methyl-2,4,5,6-tetrahydrocyclopenta[c]pyrazole* (**2-1a**). ^1^H-NMR (400 MHz, DMSO) δ 7.59 (m, 3H), 2.63 (t, *J* = 7.4, 2H), 2.56 (t, *J* = 7.1, 2H), 2.39–2.33 (m, 2H), 2.33 (s, 3H). ^13^C-NMR (126 MHz, DMSO) δ 149.31, 144.74, 141.60, 134.89, 129.22, 124.16, 116.20, 30.39, 26.13, 21.65, 12.50 (s). MS (DCI+) *m/z* 267.1 [M+H]^+^. 8% yield. In the ROESY spectrum an NOE correlation was observed between the methyl protons and resonances for the aryl ringverifying the regioisomer.


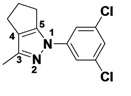

*1-(3,5-Dichlorophenyl)-3-methyl-1,4,5,6-tetrahydrocyclopenta[c]pyrazole* (**2-1b**). ^1^H-NMR (400 MHz, DMSO) δ 7.57 (d, *J* = 1.8, 2H), 7.45 (t, *J* = 1.8, 1H), 3.05 (m, 2H), 2.59–2.49 (m, 4H), 2.16 (s, 3H). MS (DCI+) *m/z* 267.1 [M+H]^+^. 1% yield. In the ROESY spectrum NOE correlations were observed between methylene protons of the cyclopentyl ring and aryl protons verifying the regioisomer.


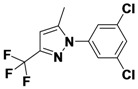

*1-(3,5-Dichlorophenyl)-5-methyl-3-(trifluoromethyl)-1H-pyrazole* (**2-2**). ^1^H-NMR (400 MHz, DMSO) δ 7.84 (dt, *J* = 16.5, 1.8, 1H), 7.76 (d, *J* = 1.8, 2H), 6.81 (s, 1H), 2.40 (s, 3H). ^13^C-NMR (126 MHz, DMSO) δ 151.34, 144.72, 133.82, 123.92 (q, *J* = 287), 119.10, 114.40, 92.23 (q, *J* = 32), 47.70, 15.31. MS (DCI+) *m/z* 294.1 [M+H]^+^. 15% yield. In the ROESY spectrum an NOE correlation was observed between the methyl protons and aryl protons, verifying the regioisomer.


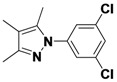

*1-(3,5-Dichlorophenyl)-3,4,5-trimethyl-1H-pyrazole* (**2-3**). ^1^H-NMR (400 MHz, DMSO) δ 7.67–7.43 (m, 3H), 2.27 (s, 3H), 2.12 (s, 3H), 1.92 (s, 3H). ^13^C-NMR (101 MHz, DMSO) δ 148.64, 141.80, 136.25, 134.31, 125.69, 121.70, 114.16, 11.66, 10.83, 7.82. MS (DCI+) *m/z* 255.1 [M+H]^+^. 26% yield.


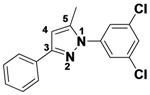

*1-(3,5-Dichlorophenyl)-5-methyl-3-phenyl-1H-pyrazole* (**2-4a**). ^1^H-NMR (400 MHz, DMSO) δ 7.85 (dt, *J* = 8.2, 1.7, 2H), 7.75 (d, *J* = 1.8, 2H), 7.70 (t, *J* = 1.8, 1H), 7.38–7.32 (m, 2H), 6.81 (d, *J* = 0.8, 1H), 2.45 (d, *J* = 0.5, 3H). ^13^C-NMR (126 MHz, DMSO) δ 149.74, 143.53, 141.46, 134.02, 129.64, 128.74, 128.52, 126.39, 122.90, 109.03, 13.22. MS (DCI+) *m/z* 303.2 [M+H]^+^. 14% yield. In the ROESY spectrum an NOE correlation was observed between the methyl protons and aryl protons of the chlorinated ring, verifying the regioisomer.


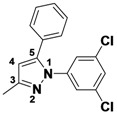

*1-(3,5-Dichlorophenyl)-3-methyl-5-phenyl-1H-pyrazole* (**2-4b**). ^1^H-NMR (400 MHz, DMSO) δ 7.57 (t, *J* = 1.9, 1H), 7.47–7.36 (m, 3H), 7.32–7.25 (m, 3H), 7.26 (d, *J* = 0.6, 1H), 6.48 (s, 1H), 2.24 (d, *J* = 34.1, 3H) MS (DCI+) *m/z* 303.2 [M+H]^+^. 9% yield. In the ROESY spectrum NOE correlations were observed between the aryl resonances for the chlorinated ring and the phenyl ring, verifying the regioisomer.


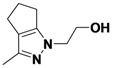

*2-(3-Methyl-5,6-dihydrocyclopenta[c]pyrazol-1(4H)-yl)ethanol* (**2-5**). ^1^H-NMR (400 MHz, DMSO) δ 3.94 (t, *J* = 5.6, 2H), 3.63 (t, *J* = 5.6, 2H), 2.70–2.65 (m, 2H), 2.48–2.40 (m, 4H), 2.07 (s, 3H). ^13^C-NMR (126 MHz, DMSO) δ 153.97, 139.13, 124.94, 76.27, 59.47, 52.43, 29.83, 23.75, 22.20, 11.08. MS (DCI+) *m/z* 167.2 [M+H]^+^. 50% yield. In the ROESY spectrum NOE correlations were observed between methylene protons in the cyclopentyl ring and the methylene protons for the alcohol substituent, verifying the regioisomer.


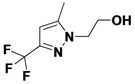

*2-(5-Methyl-3-(trifluoromethyl)-1H-pyrazol-1-yl)ethanol* (**2-6**). ^1^H-NMR (400 MHz, DMSO) δ 6.45 (s, 1H), 4.92 (t, *J* = 5.4, 1H), 4.21–4.08 (m, 2H), 3.71 (q, *J* = 5.5, 2H), 2.30 (dd, *J* = 17.1, 10.9, 3H). ^13^C-NMR (101 MHz, DMSO) δ 141.76, 139.76 (q, *J* = 37), 121.99 (q, *J* = 268), 103.30, 60.33, 51.90, 10.78. MS (DCI+) *m/z* 195.1 [M+H]^+^. 17% yield. In the ROESY spectrum NOE correlations were observed between methyl protons and methylene protons for the alcohol substituent, verifying the regioisomer.


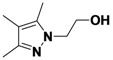

*2-(3,5-Dimethyl-1H-pyrazol-1-yl)ethanol* (**2-7**). ^1^H-NMR (400 MHz, DMSO) δ 4.06 (t, *J* = 5.6, 2H), 3.64 (t, *J* = 5.6, 2H), 2.18 (s, 3H), 2.11 (s, 3H), 1.90–1.82 (s, 3H). ^13^C-NMR (126 MHz, DMSO) δ 143.46, 139.36, 111.58, 59.87, 50.65, 10.55, 9.19, 7.44. MS (DCI+) *m/z* 155.1 [M+H]^+^. 37% yield.


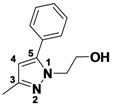

*2-(3-Methyl-5-phenyl-1H-pyrazol-1-yl)ethanol* (**2-8a**). ^1^H-NMR (400 MHz, DMSO) δ 7.64–7.27 (m, 5H), 6.15 (s, 1H), 4.03 (t, *J* = 5.9, 2H), 3.75 (t, *J* = 5.9, 2H), 2.20 (s, 3H). ^13^C-NMR (126 MHz, DMSO) δ 146.50, 144.43, 130.42, 128.81, 128.70, 128.38, 105.35, 59.98, 50.84, 13.28. MS (DCI+) *m/z* 203.1 [M+H]^+^. 57% yield. In the ROESY spectrum NOE correlations were observed between resonances for the phenyl ring and methylene protons of the alcohol substituent, verifying the regioisomer.


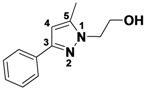

*2-(5-Methyl-3-phenyl-1H-pyrazol-1-yl)ethanol* (**2-8b**). ^1^H-NMR (400 MHz, DMSO) δ 7.73 (dd, *J* = 8.3, 1.2, 2H), 7.44–7.31 (m, 2H), 7.25 (ddd, *J* = 8.6, 2.6, 1.3, 1H), 6.44 (s, 1H), 4.09 (t, *J* = 5.8, 2H), 3.72 (t, *J* = 5.8, 2H), 2.30 (s, 3H). MS (DCI+) *m/z* 203.1 [M+H]^+^. 22% yield. In the ROESY spectrum NOE correlations were observed between methyl protons and methylene protons of the alcohol substituent, verifying the regioisomer.


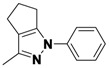

*3-Methyl-1-phenyl-1,4,5,6-tetrahydrocyclopenta[c]pyrazole* (**2-9**). ^1^H-NMR (400 MHz, DMSO) δ 7.59 (d, *J* = 8.8, 2H), 7.51–7.34 (m, 2H), 7.31–7.14 (m, 1H), 3.10–2.91 (m, 2H), 2.15 (s, 3H). ^13^C-NMR (126 MHz, DMSO) δ 148.68, 143.03, 139.94, 129.43, 127.94, 125.03, 118.03, 30.53, 26.25, 21.76, 12.53. MS (DCI+) *m/z* 199.2 [M+H]^+^. 32% yield. In the ROESY spectrum NOE correlations were observed between methylene protons for the cyclopentyl ring and resonances for the phenyl ring, verifying the regioisomer.


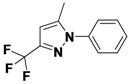

*5-Methyl-1-phenyl-3-(trifluoromethyl)-1H-pyrazole* (**2-10**). ^1^H-NMR (400 MHz, DMSO) δ 7.55 (m, 5H), 6.76 (s, 1H), 2.36 (s, 3H). ^13^C-NMR (101 MHz, DMSO) δ 141.57, 140.99 (q, *J* = 37), 138.42, 129.34, 128.75, 125.03, 121.56 (q, *J* = 268), 104.90 (q, *J* = 2), 11.84.MS (DCI+) *m/z* 227.2 [M+H]^+^. 38% yield. In the ROESY spectrum an NOE correlation was observed between methyl protons and resonances for the phenyl ring verifying the regioisomer.


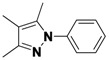

*3,4,5-Trimethyl-1-phenyl-1H-pyrazole* (**2-11**). ^1^H-NMR (400 MHz, DMSO) δ 7.53–7.40 (m, 4H), 7.40–7.26 (m, 1H), 2.20 (s, 3H), 2.13 (s, 3H), 1.93 (m, 3H). ^13^C-NMR (126 MHz, DMSO) δ 147.03, 139.75, 135.83, 129.05, 126.78, 124.01, 112.89, 11.65, 10.75, 7.90. MS (DCI+) *m/z* 187.3 [M+H]^+^. 33% yield.


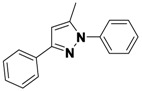

*5-Methyl-1,3-diphenyl-1H-pyrazole* (**2-12**) ^1^H-NMR (400 MHz, DMSO) δ 7.46–7.26 (m, 6H), 7.27–7.13 (m, 3H), 6.45 (s, 1H), 2.25 (s, 3H). ^13^C-NMR (126 MHz, DMSO) δ 148.53, 143.11, 139.86, 130.29, 128.93, 128.54, 128.32, 128.19, 127.23, 124.96, 107.80, 13.27. MS (DCI+) *m/z* 235.3 [M+H]^+^. 29% yield. In the ROESY spectrum an NOE correlation was observed between the methyl protons and resonances for the N-bound phenyl ring, verifying the regioisomer.


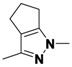

*1,3-Dimethyl-1,4,5,6-tetrahydrocyclopenta[c]pyrazole* (**2-13**). ^1^H-NMR (400 MHz, DMSO) δ 3.57 (s, 1H), 2.59 (m, 1H), 2.42 (m, 1H), 1.98 (m, 1H). ^13^C-NMR (126 MHz, DMSO) δ 150.13, 139.74, 123.91, 36.29, 30.69, 23.10, 22.51, 12.39. MS (DCI+) *m/z* 137.1 [M+H]^+^. 43% yield. In the ROESY spectrum NOE correlations were observed between methylene resonances for the cyclopentyl ring and the N-bound methyl group, verifying the regioisomer.


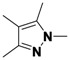

*1,3,4,5-Tetramethyl-1H-pyrazole* (**2-15**). ^1^H-NMR (400 MHz, DMSO) δ 3.57 (s, 3H), 2.08 (s, 3H), 1.99 (s, 3H), 1.82 (s, 3H). ^13^C-NMR (126 MHz, DMSO) δ 143.96, 135.34, 110.06, 35.45, 11.50, 9.05, 7.85. MS (DCI+) *m/z* 125.2 [M+H]^+^. 44% yield.


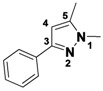

*1,5-Dimethyl-3-phenyl-1H-pyrazole* (**2-16a**). ^1^H-NMR (400 MHz, DMSO) δ 7.72 (dt, *J* = 8.1, 1.6, 2H), 7.41–7.31 (m, 2H), 7.31–7.17 (m, 1H), 6.46 (s, 1H), 3.75 (s, 3H), 2.25 (s, 3H). ^13^C-NMR (126 MHz, DMSO) δ 146.04, 143.51, 130.31, 128.79, 128.33, 128.32, 105.28, 37.07, 13.14. MS (DCI+) *m/z* 173.1 [M+H]^+^. 15% yield. In the ROESY spectrum an NOE correlation was observed between the two methyl groups, verifying the regioisomer.


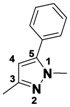

*1,3-Dimethyl-5-phenyl-1H-pyrazole* (**2-16b**). ^1^H-NMR (400 MHz, DMSO) δ 7.61–7.27 (m, 5H), 6.18 (s, 1H), 3.76 (s, 3H), 2.20 (s, 3H). MS (DCI+) *m/z* 173.1 [M+H]^+^. 6% yield. In the ROESY spectrum an NOE correlation was observed between resonances for the phenyl ring and the N-bound methyl group, verifying the regioisomer.


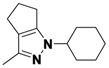

*1-Cyclohexyl-3-methyl-1,4,5,6-tetrahydrocyclopenta[c]*pyrazole (**2-17**). ^1^H-NMR (500 MHz, DMSO) δ 4.02-3.85 (m, 2H), 2.78–2.73 (m, 2H), 2.49–2.36 (m, 4H), 1.94 (s, 3H), 2.0–1.87 (m, 2H), 1.82–1.75 (m, 2H), 1.63 (qd, *J* = 12.5, 3.5, 3H), 1.35 (qt, *J* = 13.1, 3.4, 2H), 1.17 (qt, *J* = 13.0, 3.6, 1H). ^13^C-NMR (101 MHz, DMSO) δ 151.43, 138.86, 125.16, 59.77, 31.89, 29.81, 24.80, 24.60, 24.41, 21.64, 11.14. MS (DCI+) *m/z* 205.3 [M+H]^+^. 40% yield. In the ROESY spectrum NOE correlations were observed between methylene resonances for the cyclopentyl ring and resonances for the cyclohexyl substituent, verifying the regioisomer.


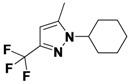

*1-Cyclohexyl-5-methyl-3-(trifluoromethyl)-1H-*pyrazole (**2-18**). ^1^H-NMR (400 MHz, DMSO) δ 6.43 (s, 1H), 4.18 (tt, *J* = 11.5, 3.9, 1H), 2.32 (s, 3H), 1.85–1.60 (m, 8H), 1.48–1.34 (m, 2H), 1.28–1.14 (m, 1H). ^13^C-NMR (101 MHz, DMSO) δ 139.66, 138.99 (q, *J* = 37), 121.81 (q, *J* = 268), 103.08 (q, *J* = 2), 57.00, 32.26, 24.81, 24.75, 10.34. MS (DCI+) *m/z* 233.3 [M+H]^+^. 23% yield. In the ROESY spectrum NOE correlations were observed between methyl protons and resonances for the cyclohexyl substituent, verifying the regioisomer.


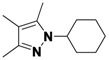

*1-Cyclohexyl-3,4,5-trimethyl-1H-pyrazole* (**2-19**). ^1^H-NMR (400 MHz, DMSO) δ 4.11–3.86 (m, 1H), 2.16 (s, 3H), 2.08 (s, 3H), 1.85 (s, 3H), 1.82–1.57 (m, 7H), 1.50–1.27 (m, 2H), 1.26–1.02 (m, 1H). ^13^C-NMR (101 MHz, DMSO) δ 143.58, 137.52, 111.25, 56.69, 32.05, 25.01, 24.81, 10.81, 9.05, 7.43. MS (DCI+) *m/z* 193.2[M+H]^+^. 34% yield.


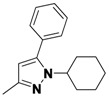

*1-Cyclohexyl-3-methyl-5-phenyl-1H-pyrazole* (**2-20**). ^1^H-NMR (400 MHz, DMSO) δ 7.57–7.27 (m, 5H), 6.06 (s, 1H), 3.98 (tt, *J* = 11.2, 4.2, 1H), 2.19 (s, 3H), 2.00–1.68 (m, 6H), 1.52–1.50 (m, 1H), 1.36 –1.01 (m, 3H). ^13^C-NMR (101 MHz, DMSO) δ 146.10, 142.90, 130.66, 128.88, 128.66, 128.42, 105.20, 56.81, 33.04, 25.15, 24.88, 13.44. MS (DCI+) *m/z* 241.2 [M+H]^+^. 26% yield. In the ROESY spectrum an NOE correlation was observed between resonances for the phenyl ring and resonances for the cyclohexyl substituent, verifying the regioisomer.

## 4. Conclusions

In this paper we have demonstrated a new method for the production of compound libraries in a continuous flow reactor using immiscible solvent spacers. The yields for compound libraries synthesized using this technique were consistent with that of standard microwave conditions. Importantly, because of the continuous nature of this methodology, the 20 member libraries took 45 min to complete in flow, but would have taken 100 min to complete if synthesized sequentially in a microwave reactor. For larger libraries the time savings using this system quickly becomes significant. Furthermore, the continuous flow setup offers the possibility of a tandem arrangement with a variety of analytical or biological techniques which could improve efficiency even further. Library synthesis in this format also uses a very small amount of organic solvent, with all forty compounds discussed here synthesized using a total of eight mL of DMF, with all of the fluorinated spacer solvent being recycled for further library synthesis.

Future studies are directed toward automating reagent loading and injection to enable larger library production in an automated fashion. We also intend to explore coupling the flow system directly to an analytical device for real time library monitoring and methodology development.

## References

[1] Tarleton M., McClusky A. (2011). A flow chemistry route to 2-phenyl-3-(1*H*-pyrrol-2-yl)propan-1-amines. Tetrahedron Lett..

[2] Castellano S., Tamborini L., Viviano M., Pinto A., Sbardella G., Conti P.  (2010). Synthesis of 3-aryl/benzyl-4,5,6,6a-tetrahydro-3aH-pyrrolo[3,4-d]isoxazole derivatives: A comparison between conventional. Microwave-assisted and flow-based methodologies. J. Org. Chem..

[3] Tanaka K., Fukase K. (2009). Renaissance of traditional organic reactions under microfluidic conditions: A new paradigm for natural product synthesis. Org. Proc. Res. Dev..

[4] Riva E., Rencurosi A., Gagliardi S., Passarella D., Martinelli M. (2011). Synthesis of (+)-dumetorine and congeners by using flow chemistry technologies. Chem. Eur. J..

[5] Achanta S., Liautard V., Paugh R., Organ M.G. (2010). The development of a general strategy for the synthesis of tyramine-based natural products by using continuous flow techniques. Chem. Eur. J..

[6] Baumann M., Baxendale I.R., Brasholz M., Hayward J.J., Ley S.V., Nikbin N. (2011). An integrated flow and batch-based approach for the synthesis of *O*-methyl siphonazole. SynLett.

[7] Shestopalov I., Tice J.D., Ismagilov R.F.  (2004). Multi-step synthesis of nanoparticles performed on millisecond time sale in a mircrofluidic droplet-based system. Lab Chip.

[8] Li S., Xu J., Wang Y., Luo G. (2008). Controllable preparation of nanoparticles by drops and plugs flow in a microchannel device. Langmuir.

[9] Urbant P., Leshansky A., Halupovich Y. (2008). On the forced convective heat transport in a droplet-laden flow in microshannels. Microfluid Nanofluid.

[10] Panke G., Schwalbe T., Stirner W., Taghavei-Moghadam S., Wille G. (2003). A practical approach of continuous processing to high energetic nitration. Synthesis.

[11] Kulkarni A.A., Kalyani V.S., Joshi R.A., Joshi R.R. (2009). Continuous flow nitration of benzaldehyde. Org. Process Res. Dev..

[12] Brocklehurst C.E., Lehmann H., Vecchia L.L.  (2011). Mitration chemistry in continuous flow using fuming nitric acid in a commercially available flow reactor. Org. Process Res. Dev..

[13] Wheeler R.C., Benali O., Deal M., Farrant E., MacDonald S.J.F., Warrington B.H. (2007). Mesoscale flow chemistry: A plug flow approach to reaction optimization. Org. Proc. Res. Dev..

[14] Song H., Tice J.D., Ismagilov R.F. (2003). A microfluidic system for controlling reaction networks in time. Angrew. Chem. Int. Ed..

[15] Zheng B., Roach S., Ismagilov R.F. (2003). Screening of protein crystallization conditions on a microfluidic chip using nanolinter-size droplets. J. Am. Chem. Soc..

[16] Song H., Ismagilov R.F. (2003). Millisecond kinetics on a microfluidic chip using nanoliters of reagents. J. Am. Chem. Soc..

[17] Zheng B., Ismagilov R.F. (2005). A Microfluidic approach for screening submicroliter volumes against multiple reagents by using preformed arrays of nanoliter plugs in a three-phase liquid/liquid/gas flow. Angrew. Chem. Int. Ed..

[18] Casadevall I Sovas X., deMello A. (2011). Droplet microfluidics: recent developments and future applications. Chem. Commun..

[19] Song H., Chen D.L., Ismagilov R. (2006). Reactions in droplets in microfluidic channels. Angrew. Chem. Int. Ed..

[20] Uniqsis Accessible Flow Chemistry. http://www.uniqsis.com/.

